# Cannabis‐Based Medicinal Products for Endometriosis: A 2‐Year Prospective Analysis From the UK Medical Cannabis Registry

**DOI:** 10.1111/ajo.70173

**Published:** 2026-08-11

**Authors:** Tania Ahmed, Simon Erridge, Evonne Clarke, Katy McLachlan, Ross Coomber, Shelley Barnes, Alia Darweish Medniuk, Rahul Guru, Wendy Holden, Mohammed Sajad, Robert Searle, Azfer Usmani, James J. Rucker, Michael Platt, Mikael H. Sodergren

**Affiliations:** ^1^ Medical Cannabis Research Group Imperial College London London UK; ^2^ Curaleaf Clinic London UK; ^3^ St. George's Hospital NHS Trust London UK; ^4^ North Bristol NHS Trust Bristol UK; ^5^ Cardiff and Vale University Health Board Cardiff UK; ^6^ Department of Psychological Medicine Kings College London London UK; ^7^ South London & Maudsley NHS Foundation Trust London UK

**Keywords:** adverse events, cannabis, pain in endometriosis, quality of life

## Abstract

**Background:**

Endometriosis affects up to 10% of biological females of reproductive age. Current treatment options are limited and often unsuitable for prolonged use. Cannabis‐based medicinal products (CBMPs) have emerged as an alternative for pain management.

**Aims:**

To analyse changes in patient‐reported outcome measures (PrOMs), prescribed opioid burden, and the prevalence of adverse events (AEs) in patients prescribed CBMPs for endometriosis‐associated pain.

**Materials and Methods:**

This was an observational analysis of prospectively collected data from the UK Medical Cannabis Registry. Biological females (≥ 18 years) with a primary diagnosis of endometriosis, enrolled ≥ 2 years prior to data extraction on 06/01/2025, were included. PrOMs and prescribed oral morphine equivalents (OME) were assessed between baseline and 1, 3, 6, 12, 18, and 24 months. Changes from baseline were assessed by repeated‐measures ANOVA and Bonferroni‐adjusted post hoc pairwise t‐tests. *p* < 0.050 was considered statistically significant.

**Results:**

One hundred and one patients were included. Improvements from baseline were observed in BPI Severity, BPI Interference, SF‐MPQ‐2 Total, Pain VAS, EQ‐5D‐5L Index, GAD‐7, and SQS at all follow‐ups (*p* < 0.001). Mean prescribed OME decreased from 19.9 ± 17.2 mg/day at baseline to 14.8 ± 15.9 mg/day at 24 months. Eighteen participants (17.8%) reported 165 AEs, of which 84 (50.9%) were mild. The most frequent were fatigue (*n* = 16; 15.8%), lethargy (*n* = 15; 14.9%), and headache (*n* = 13; 12.9%).

**Conclusion:**

CBMP treatment was associated with sustained improvements in pain, health‐related quality of life, sleep, and anxiety at 24 months, with a favourable AE profile. Randomised controlled trials are required to establish efficacy and safety.

## 1 Introduction

Endometriosis is a chronic condition characterised by endometrial tissue outside the uterus [1]. It affects up to 10% of females of reproductive age, often causing significant physical and emotional distress [1]. Symptoms include dysmenorrhoea, chronic pelvic pain, dyspareunia, and in some cases, infertility due to ovarian involvement [1].

Management involves non‐steroidal anti‐inflammatory drugs (NSAIDs) [1] and opioids [2]. While NSAIDs may provide symptom relief, evidence supporting their efficacy in endometriosis‐related pain is limited [3]. Furthermore, they are not suitable for long‐term use due to cardiovascular and renal risks [3]. Opioids carry additional risks of tolerance, addiction, and opioid‐induced hyperalgesia [4]. Hormonal therapies are often used to reduce the symptoms related to cyclical growth and shedding of ectopic endometrial tissue [5]. While effective, these therapies are not suitable for patients attempting to conceive and may not adequately address non‐cyclical pain [6]. Surgical excision is invasive and carries risks including infection and prolonged recovery [7].

Cannabis‐based medicinal products (CBMPs) have emerged as an alternative for managing endometriosis‐associated chronic pain. CBMPs include phytocannabinoids such as cannabidiol (CBD) and Δ9‐tetrahydrocannabinol (THC), which interact with the endocannabinoid system through cannabinoid receptor type 1 (CB_1_) and type 2 (CB_2_) [8, 9]. Anandamide, an endogenous cannabinoid, binds to these receptors to modulate pain [8, 9]. THC is a partial agonist of CB_1_ and CB_2_, while CBD increases anandamide by inhibiting its breakdown [8, 9].

In rodent models of inflammatory pain, CBD has demonstrated immunomodulatory and analgesic effects, including improved weight bearing and reduced oedema [10]. In the same study, THC reduced pain sensitivity [10]. CBD has also reduced neuropathic pain in rat models of neuropathy and chronic inflammation [11], while THC reversed pain hypersensitivity in surgically induced endometriosis models [12].

However, there is insufficient clinical evidence to support routine use of cannabis‐based medicinal products (CBMPs) for endometriosis‐associated pain. Two meta‐analyses across chronic pain aetiologies report modest reductions in severity with CBMPs, with one reporting a 10% risk difference for achieving a minimal clinically important difference (MCID) compared to placebo, on low‐certainty evidence [13, 14]. Conclusions were based on low‐certainty evidence from trials with high risks of bias [13, 14]. A recent scoping review by our group highlights there are no available randomised controlled trials examining CBMPs for endometriosis‐associated pain [15]. Our group previously published interim analysis of the UK Medical Cannabis Registry (UKMCR) on 63 women with endometriosis showing improved pain severity up to 18 months [16]. This study aims to extend that analysis through analysis of the changes in pain‐specific and other patient‐reported outcome measures (PrOMs), adverse event (AE) incidence, and change in prescribed opioids up to 24 months in a larger cohort.

## 2 Materials and Methods

### 2.1 Overall Design

This cohort study analysed prospectively collected UKMCR data. The cohort, inclusion criteria, and analytic plan were defined retrospectively at the point of data extraction. PrOMs, prescription details, and adverse events were collected prospectively at pre‐specified timepoints from baseline to 24 months using validated instruments.

### 2.2 Participants

Females (≥ 18 years) with a primary diagnosis of endometriosis were eligible. Inclusion criteria included UKMCR enrolment for at least 2 years prior to data extraction (06/01/2025) and completion of at least one baseline questionnaire. The UKMCR was established in December 2019 and the included participants enrolled in the registry between 01/12/2019 and 06/01/2023. All participants provided informed consent. The primary indication was recorded by the prescribing clinician at the point of enrolment in the UKMCR, with confirmation from medical records. Participants in this analysis include those reported in a prior interim analysis of the first 63 women enrolled with endometriosis; however the inclusion period and follow up is extended in the present study [16]. Ethical approval was granted by the Central Bristol Research Ethics Committee (Ref: 22/SW/0145).

### 2.3 Procedure

Patients were prescribed CBMPs in line with UK guidance. CBMPs were legalised in the UK in November 2018 for use in patients with a clear clinical need, where licensed treatments have been ineffective [17]. Product formats included ointment (administered topically), dried flower or vape cartridges (vaporised or inhaled), oils (administered sublingually), or pastilles (administered orally). When a dosing range was indicated, the midpoint was used.

PrOMs were completed remotely by participants through a secure web‐based patient portal at baseline and at 1, 3, 6, 12, 18, and 24 months. Follow‐up appointments were also conducted. AEs were reported by patients or clinicians, either remotely or during follow‐up.

### 2.4 Data Collection

Baseline demographics, indication for treatment, smoking status, weekly alcohol consumption and current or previous cannabis consumption were also recorded.

Product combinations and prescription formulations, detailing the THC and CBD concentrations, were reported throughout.

#### 2.4.1 Patient‐Reported Outcome Measures

The Brief Pain Inventory short form (BPI) is an 11‐item questionnaire, measuring pain severity (BPI Severity) and interference (BPI Interference) [18]. Participants rate each item on a scale from zero to 10, with higher scores reflecting increased severity or interference [18]. The MCID of the BPI is a 1‐point improvement [19].

The Short Form McGill Pain Questionnaire‐2 (SF‐MPQ‐2) measures the intensity and quality of pain [18]. It consists of 22 descriptors of pain in categories including: continuous, intermittent and neuropathic pain [18]. Each descriptor is rated from zero (no pain) to 10 (worst pain ever during the past week) [18]. The total score is the mean of all 22 descriptors [18]. The MCID is a 1‐point or more improvement [19].

The Pain Visual Analogue Scale (Pain VAS) is a pain measure, using a scale starting from “no pain” to “worst pain” [19]. The total length of the scale measures 10 cm, and the score is derived from how far along the line each patient marks their pain intensity [19]. The MCID is a change of 1cm [19].

The Patient Global Impression of Change (PGIC) is a single‐item measurement of how a patient's condition has changed from baseline [20]. It is the only PrOM not recorded at baseline. Each participant chooses one of seven options on a scale between one (no change) to seven (a great deal better) [20].

The European Quality of Life 5‐Dimensional Questionnaire 5‐Level Version (EQ‐5D‐5L) evaluates five parameters: mobility, self‐care, usual activities, pain/discomfort, and anxiety/depression [21]. Participants rank each of these parameters from one (no problems) to five (extreme problems/unable to do). From this a UK‐specific overall index score is derived, with a maximum score of one [21].

The Generalised Anxiety Disorder seven‐item scale (GAD‐7) has 7 items describing different symptoms of anxiety that participants rank based on how often they experience the symptom, from zero (not at all) to three (nearly every day) [22]. The total score is the sum of all items, up to 21. The MCID is a reduction of 4 or more points [22].

The Single‐Item Sleep Quality Scale (SQS) rates sleep quality over a one‐week recall period, ranging from zero (terrible) to 10 (excellent) [23]. The MCID is an improvement of 2.6 or more [23].

#### 2.4.2 Adverse Events

AEs were reported remotely or during follow‐ups. They were classified as mild, moderate, severe, or life‐threatening/disabling, per the Common Terminology Criteria for Adverse Events version 4.0 [24].

#### 2.4.3 Prescription Opioids

Prescribed opioids throughout the study period were captured and converted to an oral morphine equivalent (OME) in mg/day. A reduction of 28.2% was considered the MCID [25].

#### 2.4.4 Missing Data

Missing PrOM data were imputed using multiple imputation by chained equations with 5 imputations to maintain efficiency [26].

### 2.5 Statistical Analysis

Patient demographics, CBMP prescription formulations, and incidence of AEs were presented as the mean ± standard deviation (SD), median [interquartile range (IQR)] or frequency (percentage). Changes in PrOMs and OMEs were analysed using a repeated‐measures analysis of variance (ANOVA). Mauchly's test of sphericity was performed, and *p*‐values were corrected using the Greenhouse–Geisser method as appropriate. Post hoc pairwise *t*‐tests with Bonferroni correction were performed on statistically significant variables.

Univariable and multivariable logistic regression identified variables associated with PrOM improvement or achieving MCID at 24 months. Results were reported as odds ratios (OR) and 95% confidence intervals (CI). Statistical significance was set at *p* < 0.050. Analyses were performed in R Studio (Version 2024.04.2 + 764; Posit Software, MA, USA) using R (version 4.4.3; R Core Team, Vienna, Austria).

## 3 Results

In total, 34,563 participants were identified from the UKMCR, of which 3055 (8.8%) were excluded for having no complete baseline PrOMs. From the remaining 31,508 (91.2%), participants enrolled ≥ 2 years prior to data extraction (06/01/2025) and who completed at least one baseline questionnaire were included (*n* = 8,945; 28.4). Of these, 101 (1.1%) had a primary diagnosis of endometriosis.

Baseline demographics are in Table 1. The mean age and body mass index (BMI) were 35.5 ± 7.6 years and 25.9 ± 6.1 kg/m [2], respectively. The occupation “unemployed” (*n* = 31; 30.7%) and region “England” (*n* = 66; 65.4%) had the highest number of participants.

**TABLE 1:** Demographic characteristics of participants at baseline (*n* = 101).

Demographic measures	*N* (%), Mean ± SD, Median [IQR]
Sex	
Female	101 (100.0)
Age (Years)	35.5 ± 7.6
BMI (kg/m [2])	25.9 ± 6.1
Occupation	
Unemployed	31 (30.7)
Professional	23 (22.8)
Other occupations	13 (12.9)
Clerical support workers	8 (7.9)
Craft and related trades workers	6 (5.9)
Service and sales workers	6 (5.9)
Technicians and associate professionals	5 (5.0)
Managers	5 (5.0)
Elementary occupations	3 (3.0)
Government office region	
England	66 (65.4)
Scotland	24 (23.8)
Channel Islands	5 (5.0)
Wales	5 (5.0)
Other region (Single occurrence)	1 (1.0)
Weekly alcohol consumption (units)	0.0 [0.0–2.0]
Tobacco status	
Never smoked	42 (41.4)
Ex‐smoker	40 (39.6)
Current smoker	19 (18.8)
Lifetime tobacco consumption (pack‐years)	
Current smoker	10.0 [5.5–18.0]
Ex‐smoker	5.0 [1.8–15.0]
Cannabis status at baseline	
Current consumer	42 (41.6)
Ex‐consumer	30 (29.7)
Never used	29 (28.7)
Cannabis use frequency^a^	
Every day	31 (73.8)
Every other day	4 (9.5)
1–2 times per week or less	7 (16.7)
Cannabis routes^a^	
Vaporisation	26 (61.9)
Smoking	24 (57.1)
Ingestion	22 (52.4)
Topical	4 (9.5)
Lifetime cannabis consumption (gram years)	
Current consumers	4.4 [2.0–10.0]
Ex‐consumers	3.0 [1.0–5.5]

*Note:* Data represented as mean ± SD, median [IQR] or N (%) of the total number of participants included in the study. (*n* = 101).

Abbreviations: %, percentage; BMI, body mass index; N, number of participants; SD, standard deviation.

^a^ Calculated from current cannabis consumers prior to starting treatment with cannabis‐based medicinal products.

Most participants reported current (*n* = 42; 41.6%) or ex (*n* = 30; 29.7%) cannabis use prior to CBMP treatment. Median lifetime cannabis consumption before treatment was 4.4 [2.0–10.0] gram‐years in current users and 3.0 [1.0–5.5] in ex‐users.

### 3.1 Prescription Details

CBMP prescription details are presented in Table 2. The most common baseline combinations were oil alone (*n* = 33; 32.7%), dried flower and oil (*n* = 60; 59.4%), and dried flower alone (*n* = 8; 7.9%). At 24 months, oil (*n* = 22; 21.8%), and dried flower and oil (*n* = 52; 51.5%) decreased, while dried flower alone increased (*n* = 19; 18.8%).

**TABLE 2:** Prescription details from cannabis‐based medicinal products (CBMPs). The table shows the product combinations of the CBMPs taken, as well as the daily THC and CBD doses within each combination. Data represented as mean ± SD or *N* (%) of the total number of participants included in the study. (*n* = 101).

Prescription details	Baseline	1 month	3 months	6 months	12 months	18 months	24 months
Oil, *N* (%)	33 (32.7)	28 (27.7)	22 (21.8)	17 (16.8)	17 (16.8)	18 (17.8)	22 (21.8)
CBD, mean ± SD, mg/day	15.3 ± 8.4	17.4 ± 6.1	21.5 ± 10.8	23.4 ± 11.8	24.0 ± 23.1	29.5 ± 22.0	26.1 ± 21.5
THC, mean ± SD, mg/day	1.4 ± 0.7	7.5 ± 3.3	9.0 ± 4.4	12.5 ± 13.6	12.4 ± 13.7	13.1 ± 13.6	12.9 ± 12.8
Dried flower/flos, N (%)	8 (7.9)	7 (6.9)	8 (7.9)	13 (12.9)	17 (16.8)	18 (17.8)	19 (18.8)
CBD, mean ± SD, mg/day	6.4 ± 6.5	47.1 ± 32.4	59.1 ± 26.6	51.4 ± 32.6	57.4 ± 44.2	53.8 ± 43.8	62.1 ± 53.0
THC, mean ± SD, mg/day	15.9 ± 6.9	72.3 ± 42.8	97.6 ± 44.7	155.7 ± 63.2	186.1 ± 79.5	204.3 ± 86.4	200.4 ± 82.4
Dried flower/flos, Oil, *N* (%)	60 (59.4)	66 (65.4)	70 (69.3)	70 (69.3)	64 (63.4)	60 (59.4)	52 (51.5)
CBD, mean ± SD, mg/day	13.0 ± 10.2	23.5 ± 18.5	29.3 ± 23.9	29.0 ± 22.7	34.8 ± 27.4	35.4 ± 27.4	38.0 ± 30.8
THC, mean ± SD, mg/day	22.6 ± 6.5	135.4 ± 51.5	152.44 ± 57.8	154.5 ± 59.2	183.7 ± 84.9	175.9 ± 86.4	170.8 ± 83.9
Other	0 (0)	0 (0)	1 (1.0)	1 (1.0)	3 (3.0)	5 (5.0)	8 (8.0)

Abbreviations: CBD, Cannabidiol; NA, not applicable; *N*, number of participants; %, percentage; SD, standard deviation; THC, (−)‐trans‐Δ [9]‐tetrahydrocannabinol.

Across all products, CBD concentration was 13.3 ± 9.6 mg/day at baseline and 42.4 ± 38.0 mg/day at 24 months. THC concentration was 15.2 ± 11.2 mg/day at baseline and 143.8 ± 100.9 mg/day at 24 months.

### 3.2 Prescribed Opioids

At baseline the mean prescribed daily OME was 19.9 ± 17.2 mg/day. By 24 months this had reduced to 14.8 ± 15.9 mg/day (Tables S1 and S2). At 24 months 26.1% of participants (12/46 prescribed opioids at any point) reported the MCID in OME reduction.

### 3.3 Patient‐Reported Outcome Measures

Mean PrOM scores at each follow‐up are presented in Table 3. Differences across time were observed in mean EQ‐5D‐5L Index, GAD‐7, SQS, BPI Severity, BPI Interference, SF‐MPQ‐2 Total, and Pain VAS (*p* < 0.001). On post hoc pairwise comparison, BPI Severity (*p* < 0.001), BPI Interference (*p* < 0.010), SF‐MPQ‐2 Total (*p* < 0.001), and Pain VAS (*p* < 0.050) decreased from baseline at all subsequent follow‐ups (Figure S1). Full pairwise analysis of all PrOMs and the proportion of individuals who reported MCID in each are detailed in Tables S3–S18.

**TABLE 3:** Mean patient‐reported outcome measure (PrOM) scores for each questionnaire from baseline up to 24 months. (*n* = 101). Repeated measures analysis of variance (ANOVA) to compare PrOMs. For each outcome, sphericity assumptions were evaluated using Mauchly's test, with Greenhouse–Geisser corrections applied when sphericity was violated.

PrOM Questionnaires	Mean ± SD							*p*
	Baseline	1 month	3 months	6 months	12 months	18 months	24 months	
EQ‐5D‐5L mobility	2.3 ± 1.1	2.2 ± 1.2	2.2 ± 1.1	2.3 ± 1.2	2.2 ± 1.3	2.2 ± 1.3	1.9 ± 0.9	0.065
EQ‐5D‐5L self‐care	1.8 ± 1.0	1.8 ± 1.1	1.7 ± 1.1	2.0 ± 1.2	1.9 ± 1.2	1.9 ± 1.0	2.0 ± 1.1	0.045
EQ‐5D‐5L usual activities	2.7 ± 1.2	2.5 ± 1.3	2.4 ± 1.4	2.5 ± 1.4	2.6 ± 1.4	2.3 ± 1.3	2.3 ± 1.1	0.035
EQ‐5D‐5L pain and discomfort	3.6 ± 1.0	2.7 ± 1.1	2.9 ± 0.9	3.0 ± 1.4	3.0 ± 1.2	2.7 ± 1.4	2.7 ± 1.1	< 0.001
EQ‐5D‐5L anxiety and depression	2.6 ± 1.1	2.2 ± 1.0	2.3 ± 1.1	2.3 ± 1.1	2.4 ± 1.2	2.1 ± 1.1	2.3 ± 1.2	0.121
EQ‐5D‐5L index	0.4 ± 0.3	0.6 ± 0.3	0.5 ± 0.3	0.5 ± 0.3	0.4 ± 0.4	0.5 ± 0.4	0.6 ± 0.3	< 0.001
GAD‐7	8.2 ± 5.9	5.7 ± 5.0	7.5 ± 6.6	5.8 ± 5.5	6.9 ± 6.2	4.4 ± 3.5	8.6 ± 7.0	< 0.001
SQS	4.8 ± 2.5	6.4 ± 2.8	6.0 ± 2.8	4.8 ± 3.3	6.1 ± 3.1	6.1 ± 3.2	5.8 ± 2.6	< 0.001
BPI severity	5.8 ± 1.6	4.8 ± 2.5	4.6 ± 2.3	4.6 ± 2.9	4.6 ± 2.6	4.2 ± 2.9	3.9 ± 2.7	< 0.001
BPI interference	6.6 ± 2.3	5.2 ± 2.9	4.5 ± 2.9	5.5 ± 3.6	4.3 ± 3.5	5.0 ± 3.9	4.5 ± 3.9	< 0.001
SF‐MPQ‐2 continuous pain subscale	6.3 ± 1.8	5.2 ± 2.6	4.6 ± 2.8	5.1 ± 3.5	5.0 ± 2.9	4.1 ± 2.9	4.7 ± 3.3	< 0.001
SF‐MPQ‐2 intermittent pain subscale	5.2 ± 2.1	3.9 ± 2.5	3.6 ± 2.6	4.5 ± 3.2	4.9 ± 3.3	3.7 ± 3.0	3.9 ± 3.2	< 0.001
SF‐MPQ‐2 neuropathic pain subscale	2.7 ± 2.1	2.3 ± 2.3	2.4 ± 2.4	2.7 ± 2.9	2.6 ± 2.5	2.1 ± 2.4	2.2 ± 2.4	0.003
SF‐MPQ‐2 total	5.1 ± 1.7	4.1 ± 2,2	3.4 ± 2.1	3.7 ± 2.9	3.5 ± 2.6	3.2 ± 2.2	3.2 ± 2.8	< 0.001
Pain VAS	6.9 ± 2.3	5.4 ± 3.0	5.4 ± 2.9	5.5 ± 3.5	5.7 ± 3.2	5.1 ± 3.6	5.4 ± 3.6	< 0.001

Abbreviations: BPI Interference, brief pain inventory interference; BPI Severity, brief pain inventory severity; EQ‐5D‐5L, European Quality of Life 5‐Dimensional Questionnaire 5‐Level Version; GAD‐7, generalised anxiety disorder‐7; SF‐MPQ‐2, Short Form McGill Pain Questionnaire‐2; Pain VAS, Pain Visual Analogue Scale; SD, standard deviation; SQS, sleep quality scale.

### 3.4 Adverse Events

Eighteen (17.8%) participants reported a total of 165 AEs (Table 4). The most frequent AEs were fatigue (*n* = 16; 15.8%), lethargy (*n* = 15; 14.9%), and headache (*n* = 13; 12.9%). The most frequent severity category was mild (*n* = 84; 50.9%). There was only one (1.0%) life‐threatening AE reported.

**TABLE 4:** Frequency of reported adverse events.

Adverse event	Mild	Moderate	Severe	Life‐threatening/disabling	Total, *N* (%)
Fatigue	3	11	2	0	16 (15.8)
Lethargy	8	7	0	0	15 (14.9)
Headache	6	6	1	0	13 (12.9)
Dry mouth	9	3	0	0	12 (11.9)
Insomnia	4	4	4	0	12 (11.9)
Somnolence	0	10	1	0	11 (10.9)
Constipation	10	0	0	0	10 (9.9)
Abdominal pain	7	2	0	0	9 (8.9)
Nausea	7	0	0	0	7 (6.9)
Dizziness	4	2	0	0	6 (5.9)
Dyspepsia	4	2	0	0	6 (5.9)
Concentration impairment	4	1	0	0	5 (5.0)
Lung infection	0	5	0	0	5 (5.0)
Pharyngitis	0	4	1	0	5 (5.0)
Urinary tract infection	0	4	0	1	5 (5.0)
Confusion	3	1	0	0	4 (4.0)
Generalised muscle weakness	1	1	2	0	4 (4.0)
Delirium	3	0	0	0	3 (4.0)
Weight loss	3	0	0	0	3 (3.0)
Anorexia	0	2	0	0	2 (2.0)
Rash NOS	2	0	0	0	2 (2.0)
Spasticity	2	0	0	0	2 (2.0)
Other, single occurrences	4	4	0	0	8 (7.9)
Total, *N* (%)	84 (83.2)	69 (68.3)	11 (10.9)	1 (1.0)	165

*Note:* Adverse events graded in accordance with the Common Terminology Criteria for Adverse Events version 4.0. % calculated a proportion of total case series (*n* = 101). Single occurrences were aggregated under ‘Other’ to avoid reidentification.

Abbreviations: NOS, not otherwise specified; N, number of participants; %, percentage.

### 3.5 Logistic Regression

On multivariable logistic regression, none of the studied variables were associated with reporting an MCID in the BPI Severity subscale (*p* > 0.050). Individuals aged under 30 were more likely to report an MCID in the BPI Interference subscale at 24 months, compared to those aged 31 to 40 (OR: 0.2; 95% CI: 0.0–0.7; *p* = 0.018), and 41 and older (OR: 0.1; 95% CI: 0.0–0.6; *p* = 0.013). Full outcomes from univariable and multivariable logistic regression are detailed in Tables S19–S26.

## 4 Discussion

Across 24 months, improvements were observed in measures of pain severity, its impact on health‐related quality of life (HRQoL), sleep, and anxiety. At 18 and 24 months, there were reductions in prescribed opioids compared to baseline, including a clinically significant reduction in 26.1% of participants at 24 months. Meanwhile, medications were well tolerated by most individuals. Eighty‐three (82.2%) patients reported no AEs. In individuals with AEs, the majority of the 165 AEs were mild (*n* = 84; 50.9%) or moderate (*n* = 69; 41.8%) in severity. These findings extend a previously reported interim analysis of 63 women by an additional 38 participants, 6 months of follow‐up, and analyses of opioid burden, severity‐stratified adverse events, and multivariable predictors of clinically meaningful response [16].

Improvements in pain severity and interference align with separate meta‐analyses by Barakji et al. and Wang et al., which examined chronic non‐cancer pain populations broadly and found moderate‐certainty evidence that CBMPs or cannabinoids provided modest improvements in pain compared to placebo [13, 14]. Neither meta‐analysis included a dedicated endometriosis subgroup, and both authors counsel caution about the role of CBMPs beyond patients who have not responded to first‐line therapy [13, 14]. A recent scoping review by our group highlights that the most common reported reason for cannabis use in endometriosis is due to pain, with many individuals reporting it effective in reducing its severity [15]. However, outside of evidence from the UKMCR, there are no studies examining a cohort of patients legally accessing CBMPs under clinical oversight. Consequently, the present analysis provides supportive evidence for sustained changes in pain severity and interference up to 2 years.

In addition to pain‐specific improvements, improvements in sleep quality and anxiety were also reported. The evidence on CBMPs in the setting of anxiety disorders and insomnia is sparse [27, 28]. However, this finding aligns with other studies of chronic pain which have incorporated secondary outcomes on anxiety, mood, or sleep, which have found improvements across all three parameters [14, 27]. Endometriosis is a condition predominantly affecting women between 20 and 50 years of age, where the prevalence of anxiety disorders is higher [29]. Moreover, there is a bidirectional effect between the development of anxiety and chronic pain [29]. Analysis beyond the direct anti‐nociceptive effects is therefore an important consideration for developing research in this field.

The adverse event rate observed in the present cohort (17.8%) lies within the range of 10.2%–52.0% reported in observational studies of cannabis use in endometriosis included in our recent scoping review [15]. The present study, however, differs from this earlier literature in several fundamental ways. Firstly, this study examines the outcomes of individuals prescribed CBMPs manufactured in line with Good Manufacturing Practice and clinical oversight. In addition, the data were collected prospectively. In comparison, prior literature captures retrospective data from a mix of medical, recreational, and illicit cannabis consumers [15].

However, limitations must be acknowledged. As an observational study, causality cannot be inferred. Selection bias is likely due to non‐randomised recruitment through a private self‐paying clinic. Patients with endometriosis enrolled in the UKMCR were entirely female, younger and were less likely to be cannabis consumers prior to initiating treatment when compared with the registry population [30]. These differences should be considered when interpreting the findings observed in the present cohort, alongside the condition of interest. The absence of a control group precludes differentiation between treatment effects, natural disease fluctuation, regression to the mean, and placebo effects. Confounding by indication is inherent to registry studies, as patients prescribed CBMPs may differ systematically from those managed with conventional therapies, particularly given prior treatment failure. Heterogeneity in CBMP formulations, doses, titration protocols, and THC:CBD ratios prevents attribution of outcomes to specific cannabinoid profiles or dosing strategies. Loss to follow‐up introduced attrition bias, with potential differential dropout between responders and non‐responders. The observational design cannot establish temporal causality between CBMP initiation and outcome changes.

In conclusion, this prospective registry study demonstrated improvements in pain, health‐related quality of life, sleep quality, and anxiety following CBMP initiation in patients with endometriosis, with benefits sustained to 24 months and a favourable adverse event profile. However, the observational design, absence of a control group, and heterogeneity in CBMP formulations preclude causal inference. These findings provide real‐world evidence supporting the potential utility of CBMPs in endometriosis‐associated pain but must be interpreted cautiously. Randomised, placebo‐controlled trials with standardised cannabinoid formulations and objective outcome measures are essential to establish efficacy, optimal dosing strategies, and long‐term safety before CBMPs can be recommended as a therapeutic option for endometriosis.

## Funding

The authors have nothing to report.

## Conflicts of Interest

Tania Ahmed is a medical student at Imperial College London. Tania Ahmed has no shareholdings in pharmaceutical companies. Simon Erridge is a resident doctor and Research Director at Curaleaf Clinic. Simon Erridge is a research fellow at Imperial College London. Simon Erridge has no shareholdings in pharmaceutical companies. Evonne Clarke is the Patient Care Director at Curaleaf Clinic. Evonne Clarke has no shareholdings in pharmaceutical companies. Katy McLachlan is the Chief Pharmacist at Curaleaf Clinic. Katy McLachlan has no shareholdings in pharmaceutical companies. Ross Coomber is a consultant orthopaedic surgeon at St George's Hospital, London, and Operations Director at Curaleaf Clinic. Ross Coomber has no shareholdings in pharmaceutical companies. Shelley Barnes is a consultant pain specialist at North Bristol NHS Trust and Curaleaf Clinic. Shelley Barnes has no shareholdings in pharmaceutical companies. Alia Darweish Medniuk is a consultant pain specialist at North Bristol NHS Trust and Curaleaf Clinic. Alia Darweish Medniuk has no shareholdings in pharmaceutical companies. Rahul Guru is a consultant pain specialist at Cardiff and Vale University Health Board and Curaleaf Clinic. Rahul Guru has no shareholdings in pharmaceutical companies. Wendy Holden is a consultant pain specialist at Curaleaf Clinic. Wendy Holden has no shareholdings in pharmaceutical companies. Mohammed Sajad is a consultant pain specialist at Curaleaf Clinic. Mohammed Sajad has no shareholdings in pharmaceutical companies. Robert Searle is a consultant pain specialist at Curaleaf Clinic. Robert Searle has no shareholdings in pharmaceutical companies. Azfer Usmani is a consultant pain specialist at Curaleaf Clinic. Azfer Usmani has no shareholdings in pharmaceutical companies. James Rucker is a consultant psychiatrist at Curaleaf Clinic. James Rucker is an honorary consultant psychiatrist at The South London & Maudsley NHS Foundation Trust, and an NIHR Clinician Scientist Fellow at the Centre for Affective Disorders at King's College London. James Rucker is funded by a fellowship (CS‐2017‐17‐007) from the National Institute for Health Research (NIHR). The views expressed are those of the author(s) and not necessarily those of the NHS, the NIHR or the Department of Health. James Rucker leads the Psychedelic Trials Group at King's College London. King's College London receives grant funding from COMPASS Pathways PLC to undertake phase 1 and phase 2 trials with psilocybin. COMPASS Pathways PLC has paid for James Rucker to attend trial related meetings and conferences to present the results of research using psilocybin. James Rucker has undertaken paid consultancy work for Beckley PsyTech and Clerkenwell Health. Payments for consultancy work are received and managed by King's College London and James Rucker does not benefit personally. James Rucker has no shareholdings in pharmaceutical companies. Michael Platt is a consultant in pain services at Curaleaf Clinic. Michael Platt has no shareholdings in pharmaceutical companies. Mikael Sodergren is a consultant hepatopancreatobiliary surgeon at Imperial College NHS Trust, London, a senior clinical lecturer at Imperial College London, and the chief medical officer of Curaleaf International. Mikael Sodergren has no shareholdings in pharmaceutical companies.

## Supporting information


**Table S1:** Comparison of Prescribed Analgesia between Baseline, 1, 3, 6, 12, 18, and 24 months.
**Table S2:** Pairwise Comparison of Prescribed Daily Oral Morphine Equivalent (mg/day).
**Table S3:** Pairwise Comparison of EQ‐5D‐5L Mobility scores.
**Table S4:** Pairwise Comparison of EQ‐5D‐5L Self‐care scores.
**Table S5:** Pairwise Comparison of EQ‐5D‐5L Usual Activities scores.
**Table S6:** Pairwise Comparison of EQ‐5D‐5L Pain and discomfort scores.
**Table S7:** Pairwise Comparison of EQ‐5D‐5L Anxiety and depression scores.
**Table S8:** Pairwise Comparison of EQ‐5D‐5L Index scores.
**Table S9:** Pairwise Comparison of GAD‐7 scores.
**Table S10:** Pairwise Comparison of SQS scores.
**Table S11:** Pairwise Comparison of BPI Severity scores.
**Table S12:** Pairwise Comparison of BPI Interference scores.
**Table S13:** Pairwise Comparison of SF‐MPQ‐2 continuous pain subscale scores.
**Table S14:** Pairwise Comparison of SF‐MPQ‐2 intermittent pain subscale scores.
**Table S15:** Pairwise Comparison of SF‐MPQ‐2 neuropathic pain subscale scores.
**Table S16:** Pairwise Comparison of SF‐MPQ‐2 Total scores
**Table S17:** Pairwise Comparison of Pain VAS scores.
**Table S18:** Proportion of individuals who reported an MCID/improvement in each PrOM at 1, 3, 6, 12, 18 and 24 months.
**Table S19:** Results of univariate logistic regression analyses evaluating factors a minimal clinically important difference in the Brief Pain Inventory‐Short Form (BPI) Severity at 24 months. Variables assessed include gender, age, body mass index (BMI), cannabis status, cannabis‐based medicinal products (CBMPs) at 24 months, cannabidiol (CBD) dose at 24 months, (−)‐trans‐Δ9‐tetrahydrocannabinol (THC) dose at 24 months, baseline Sleep Quality Scale (SQS), and baseline Generalised Anxiety Disorder‐7 (GAD‐7) scores.
**Table S20:** Results of multivariate logistic regression analyses evaluating factors a minimal clinically important difference in the Brief Pain Inventory‐Short Form (BPI) Severity at 24 months. Variables assessed include gender, age, body mass index (BMI), cannabis status, cannabis‐based medicinal products (CBMPs) at 24 months, cannabidiol (CBD) dose at 24 months, (−)‐trans‐Δ9‐tetrahydrocannabinol (THC) dose at 24 months, baseline Sleep Quality Scale (SQS), and baseline Generalised Anxiety Disorder‐7 (GAD‐7) scores.
**Table S21:** Results of univariate logistic regression analyses evaluating factors a minimal clinically important difference in the Brief Pain Inventory‐Short Form (BPI) Interference at 24 months. Variables assessed include gender, age, body mass index (BMI), cannabis status, cannabis‐based medicinal products (CBMPs) at 24 months, cannabidiol (CBD) dose at 24 months, (−)‐trans‐Δ9‐tetrahydrocannabinol (THC) dose at 24 months, baseline Sleep Quality Scale (SQS), and baseline Generalised Anxiety Disorder‐7 (GAD‐7) scores.
**Table S22:** Results of multivariate logistic regression analyses evaluating factors a minimal clinically important difference in the Brief Pain Inventory‐Short Form (BPI) Interference at 24 months. Variables assessed include gender, age, body mass index (BMI), cannabis status, cannabis‐based medicinal products (CBMPs) at 24 months, cannabidiol (CBD) dose at 24 months, (−)‐trans‐Δ9‐tetrahydrocannabinol (THC) dose at 24 months, baseline Sleep Quality Scale (SQS), and baseline Generalised Anxiety Disorder‐7 (GAD‐7) scores.
**Table S23:** Results of univariate logistic regression analyses evaluating factors a minimal clinically important difference in the Short‐Form McGill Pain Questionnaire—Version 2 (SF‐MPQ 2). Variables assessed include gender, age, body mass index (BMI), cannabis status, cannabis‐based medicinal products (CBMPs) at 24 months, cannabidiol (CBD) dose at 24 months, (−)‐trans‐Δ9‐tetrahydrocannabinol (THC) dose at 24 months, baseline Sleep Quality Scale (SQS), and baseline Generalised Anxiety Disorder‐7 (GAD‐7) scores.
**Table S24:** Results of multivariate logistic regression analyses evaluating factors a minimal clinically important difference in the Short‐Form McGill Pain Questionnaire—Version 2 (SF‐MPQ 2). Variables assessed include gender, age, body mass index (BMI), cannabis status, cannabis‐based medicinal products (CBMPs) at 24 months, cannabidiol (CBD) dose at 24 months, (−)‐trans‐Δ9‐tetrahydrocannabinol (THC) dose at 24 months, baseline Sleep Quality Scale (SQS), and baseline Generalised Anxiety Disorder‐7 (GAD‐7) scores.
**Table S25:** Results of univariate logistic regression analyses evaluating factors a minimal clinically important difference in the Visual Analogue Scale (VAS). Variables assessed include gender, age, body mass index (BMI), cannabis status, cannabis‐based medicinal products (CBMPs) at 24 months, cannabidiol (CBD) dose at 24 months, (−)‐trans‐Δ9‐tetrahydrocannabinol (THC) dose at 24 months, baseline Sleep Quality Scale (SQS), and baseline Generalised Anxiety Disorder‐7 (GAD‐7) scores.
**Table S26:** Results of multivariate logistic regression analyses evaluating factors a minimal clinically important difference in the Pain Visual Analogue Scale (VAS). Variables assessed include gender, age, body mass index (BMI), cannabis status, cannabis‐based medicinal products (CBMPs) at 24 months, cannabidiol (CBD) dose at 24 months, (−)‐trans‐Δ9‐tetrahydrocannabinol (THC) dose at 24 months, baseline Sleep Quality Scale (SQS), and baseline Generalised Anxiety Disorder‐7 (GAD‐7) scores.
**Figure S1:** Pairwise analysis of pain specific patient‐reported outcome measures (PrOMs) (*n* = 101). (A) Brief Pain Inventory—Short Form (BPI) Severity; (B) BPI Interference; (C) Short Form—McGill Pain Questionnaire—2 (SF‐MPQ‐2) Total Score; (D) Pain Visual Analogue Scale (VAS).

## Data Availability

The data that support the findings of this study are available on request from the corresponding author. The data are not publicly available due to privacy or ethical restrictions.
