# Supplementary material for: Cannabis‐Based Medicinal Products for Endometriosis: A 2‐Year Prospective Analysis From the UK Medical Cannabis Registry
Source: Aust N Z J Obstet Gynaecol. 2026 Aug 11;66(4):e70173. doi: 10.1111/ajo.70173 (PMC13458955; doi:10.1111/ajo.70173)
Supplement: Supplementary file 1 [file AJO-66-0-s001.docx]

**Supplementary Table 1. Comparison of Prescribed Analgesia between Baseline, 1, 3, 6, 12, 18, and 24 months.**

Repeated measures analysis of variance (ANOVA) to compare daily oral morphine equivalent in mg/day. For each outcome, sphericity assumptions were evaluated using Mauchly's test, with Greenhouse-Geisser corrections applied when sphericity was violated.

SD, standard deviation

|  | **Mean ± SD** | | | | | | |  |
| --- | --- | --- | --- | --- | --- | --- | --- | --- |
|  | **Baseline** | **1 month** | **3 months** | **6 months** | **12 months** | **18 months** | **24 months** | **p-value** |
| **Oral Morphine Equivalent (mg/day)** | 19.9 ± 17.2 | 20.6 ± 19.7 | 18.9 ± 19.5 | 16.0 ± 16.6 | 16.0 ± 16.6 | 15.2 ± 16.4 | 14.8 ± 15.9 | <0.001 |

**Supplementary Table 2. Pairwise Comparison of Prescribed Daily Oral Morphine Equivalent (mg/day)**

Post-hoc pairwise comparisons between time points were performed using paired t-tests with Bonferroni-Holm correction for multiple comparisons. Effect sizes were calculated using Cohen's *d* for paired differences.

SD, standard deviation

| **Follow-up Interval** | **Mean Difference ± SD, p-value** | | | | | | |
| --- | --- | --- | --- | --- | --- | --- | --- |
|  | **Baseline** | **1 month** | **3 months** | **6 months** | **12 months** | **18 months** | **24 months** |
| **Baseline** |  |  |  |  |  |  |  |
| **1 month** | 0.7 ± 8.8 p=1.000 |  |  |  |  |  |  |
| **3 months** | -1.0 ± 10.8 p=1.000 | -1.7 ± 6.0 p=0.842 |  |  |  |  |  |
| **6 months** | -3.9 ± 10.0 p=0.136 | -4.6 ± 10.9 p=0.071 | -2.9 ± 9.6 p=0.708 |  |  |  |  |
| **12 months** | -3.9 ± 10.0 p=0.136 | -4.6 ± 10.9 p=0.071 | -2.9 ± 9.6 p=0.708 | 0 ± 0 p=N/A |  |  |  |
| **18 months** | -4.6 ± 9.9 p=0.028 | -5.4 ± 14.5 p=0.202 | -3.7 ± 13.7  p=1.000 | -0.8 ± 5.6 p=1.000 | -0.8 ± 5.6 p=1.000 |  |  |
| **24 months** | -5.1 ± 10.1 p=0.015 | -5.8 ± 14.6 p=0.129 | -4.0 ± 13.9 p=0.818 | -1.2 ± 6.2 p=1.000 | -1.2 ± 6.2 p=1.000 | -0.4 ± 2.8 p=1.000 |  |

**Supplementary Table 3. Pairwise Comparison of EQ-5D-5L Mobility scores.**

Post-hoc pairwise comparisons between time points were performed using paired t-tests with Bonferroni-Holm correction for multiple comparisons. Effect sizes were calculated using Cohen's *d* for paired differences.

SD, standard deviation

| **Follow-up Interval** | **Mean Difference ± SD, p-value** | | | | | | |
| --- | --- | --- | --- | --- | --- | --- | --- |
|  | **Baseline** | **1 month** | **3 months** | **6 months** | **12 months** | **18 months** | **24 months** |
| **Baseline** |  |  |  |  |  |  |  |
| **1 month** | -0.2 ± 1.0  p=0.602 |  |  |  |  |  |  |
| **3 months** | -0.2 ± 1.3 p=1.000 | 0.1 ± 1.2 p=1.000 |  |  |  |  |  |
| **6 months** | -0.1 ± 1.5 p=1.000 | 0.1 ± 1.4 p=1.000 | 0.1 ± 1.5 p=1.000 |  |  |  |  |
| **12 months** | 0.0 ± 1.6 p=1.000 | 0.2 ± 1.5 p=1.000 | 0.2 ± 1.6 p=1.000 | 0.1 ± 1.6 p=1.000 |  |  |  |
| **18 months** | -0.2 ± 1.4 p=1.000 | 0.0 ± 1.5 p=1.000 | -0.0 ± 1.5  p=1.000 | -0.1 ± 1.7 p=1.000 | -0.2 ± 1.9 p=1.000 |  |  |
| **24 months** | -0.4 ± 1.3 p=0.030 | -0.2 ± 1.1 p=1.000 | -0.3 ± 1.3  p=1.000 | -0.3 ± 1.4 p=0.755 | -0.4 ± 1.4 p=0.040 | -0.2 ± 1.6 p=1.000 |  |

**Supplementary Table 4. Pairwise Comparison of EQ-5D-5L Self-care scores.**

Post-hoc pairwise comparisons between time points were performed using paired t-tests with Bonferroni-Holm correction for multiple comparisons. Effect sizes were calculated using Cohen's *d* for paired differences.

SD, standard deviation

| **Follow-up Interval** | **Mean Difference ± SD, p-value** | | | | | | |
| --- | --- | --- | --- | --- | --- | --- | --- |
|  | **Baseline** | **1 month** | **3 months** | **6 months** | **12 months** | **18 months** | **24 months** |
| **Baseline** |  |  |  |  |  |  |  |
| **1 month** | 0.0 ± 1.1 p=1.000 |  |  |  |  |  |  |
| **3 months** | -0.0 ± 1.2 p=1.000 | -0.0 ± 1.1 p=1.000 |  |  |  |  |  |
| **6 months** | 0.3 ± 1.3  p=0.876 | 0.2 ± 1.4 p=1.000 | 0.3 ± 1.4 p=1.000 |  |  |  |  |
| **12 months** | 0.2 ± 1.2 p=1.000 | 0.2 ± 1.3 p=1.000 | 0.2 ± 1.5 p=1.000 | -0.1 ± 1.4 p=1.000 |  |  |  |
| **18 months** | 0.1 ± 1.3 p=1.000 | 0.1 ± 1.3 p=1.000 | 0.2 ± 1.4 p=1.000 | -0.1 ± 1.4  p=1.000 | -0.0 ± 1.4 p=1.000 |  |  |
| **24 months** | 0.2 ± 1.4 p=1.000 | 0.2 ± 1.3 p=1.000 | 0.3 ± 1.4 p=1.000 | -0.0 ± 1.4 p=1.000 | 0.8 ± 1.4 p=1.000 | 0.1 ± 1.1 p=1.000 |  |

**Supplementary Table 5. Pairwise Comparison of EQ-5D-5L Usual Activities scores.**

Post-hoc pairwise comparisons between time points were performed using paired t-tests with Bonferroni-Holm correction for multiple comparisons. Effect sizes were calculated using Cohen's *d* for paired differences.

SD, standard deviation

| **Follow-up Interval** | **Mean Difference ± SD, p-value** | | | | | | |
| --- | --- | --- | --- | --- | --- | --- | --- |
|  | **Baseline** | **1 month** | **3 months** | **6 months** | **12 months** | **18 months** | **24 months** |
| **Baseline** |  |  |  |  |  |  |  |
| **1 month** | -0.2 ± 1.3  p=1.000 |  |  |  |  |  |  |
| **3 months** | -0.4 ± 1.7 p=0.980 | -0.1 ± 1.5 p=1.000 |  |  |  |  |  |
| **6 months** | -0.3 ± 1.8 p=1.000 | -0.1 ± 1.8 p=1.000 | 0.1 ± 1.8 p=1.000 |  |  |  |  |
| **12 months** | -0.1 ± 1.5 p=1.000 | 0.2 ± 1.4 p=1.000 | 0.3 ± 1.7 p=1.000 | 0.2 ± 1.9 p=1.000 |  |  |  |
| **18 months** | -0.4 ± 1.6 p=0.220 | -0.2 ± 1.7 p=1.000 | -0.1 ± 1.9 p=1.000 | -0.1 ± 1.7  p=1.000 | -0.3 ± 1.6 p=0.785 |  |  |
| **24 months** | -0.5 ± 1.7 p=0.096 | -0.2 ± 1.7 p=1.000 | -0.1 ± 1.7 p=1.000 | -0.2 ± 1.5 p=1.000 | -0.4 ± 1.7 p=0.541 | -0.1 ± 1.4 p=1.000 |  |

**Supplementary Table 6. Pairwise Comparison of EQ-5D-5L Pain and discomfort scores.**

Post-hoc pairwise comparisons between time points were performed using paired t-tests with Bonferroni-Holm correction for multiple comparisons. Effect sizes were calculated using Cohen's *d* for paired differences.

SD, standard deviation

| **Follow-up Interval** | **Mean Difference ± SD, p-value** | | | | | | |
| --- | --- | --- | --- | --- | --- | --- | --- |
|  | **Baseline** | **1 month** | **3 months** | **6 months** | **12 months** | **18 months** | **24 months** |
| **Baseline** |  |  |  |  |  |  |  |
| **1 month** | -0.9 ± 1.2 p=<0.001 |  |  |  |  |  |  |
| **3 months** | -0.7 ± 1.3 p=<0.001 | 0.2 ± 1.2 p=1.000 |  |  |  |  |  |
| **6 months** | -0.7 ± 1.6 p=0.002 | 0.3 ± 1.6 p=1.000 | 0.1 ± 1.6 p=1.000 |  |  |  |  |
| **12 months** | -0.7 ± 1.4 p=<0.001 | 0.3 ± 1.7 p=1.000 | 0.1 ± 1.5 p=1.000 | 0.0 ± 1.3 p=1.000 |  |  |  |
| **18 months** | -1.0 ± 1.6 p=<0.001 | -0.1 ± 1.7 p=1.000 | -0.2 ± 1.6 p=1.000 | -0.3 ± 1.9  p=1.000 | -0.3 ± 1.9 p=1.000 |  |  |
| **24 months** | -0.9 ± 1.5 p=<0.001 | 0.0 ± 1.5 p=1.000 | -0.2 ± 1.5 p=1.000 | -0.2 ± 1.6 p=1.000 | -0.3 ± 1.5 p=1.000 | 0.1 ± 2.0 p=1.000 |  |

**Supplementary Table 7. Pairwise Comparison of EQ-5D-5L Anxiety and depression scores.**

Post-hoc pairwise comparisons between time points were performed using paired t-tests with Bonferroni-Holm correction for multiple comparisons. Effect sizes were calculated using Cohen's *d* for paired differences.

SD, standard deviation

| **Follow-up Interval** | **Mean Difference ± SD, p-value** | | | | | | |
| --- | --- | --- | --- | --- | --- | --- | --- |
|  | **Baseline** | **1 month** | **3 months** | **6 months** | **12 months** | **18 months** | **24 months** |
| **Baseline** |  |  |  |  |  |  |  |
| **1 month** | -0.4 ± 1.2 p=0.013 |  |  |  |  |  |  |
| **3 months** | -0.3 ± 1.2  p=0.161 | 0.1 ± 1.1 p=1.000 |  |  |  |  |  |
| **6 months** | -0.3 ± 1.5 p=1.000 | 0.1 ± 1.2 p=1.000 | 0.1 ± 1.2 p=1.000 |  |  |  |  |
| **12 months** | -0.2 ± 1.5 p=1.000 | 0.2 ± 1.3 p=1.000 | 0.1 ± 1.5 p=1.000 | 0.1 ± 1.4 p=1.000 |  |  |  |
| **18 months** | -0.5 ± 1.4  p=0.028 | -0.1 ± 1.5 p=1.000 | -0.1 ± 1.4 p=1.000 | -0.2 ± 1.5 p=1.000 | -0.3 ± 1.7 p=1.000 |  |  |
| **24 months** | -0.3 ± 1.6 p=1.000 | 0.1 ± 1.6 p=1.000 | 0.1 ± 1.5 p=1.000 | -0.0 ± 1.5 p=1.000 | -0.1 ± 1.8 p=1.000 | 0.2 ± 1.4 p=1.000 |  |

**Supplementary Table 8. Pairwise Comparison of EQ-5D-5L Index scores.**

Post-hoc pairwise comparisons between time points were performed using paired t-tests with Bonferroni-Holm correction for multiple comparisons. Effect sizes were calculated using Cohen's *d* for paired differences.

SD, standard deviation

| **Follow-up Interval** | **Mean Difference ± SD, p-value** | | | | | | |
| --- | --- | --- | --- | --- | --- | --- | --- |
|  | **Baseline** | **1 month** | **3 months** | **6 months** | **12 months** | **18 months** | **24 months** |
| **Baseline** |  |  |  |  |  |  |  |
| **1 month** | 0.2 ± 0.3 p=<0.001 |  |  |  |  |  |  |
| **3 months** | 0.2 ± 0.3 p=0.001 | -0.0 ± 0.3  p=1.000 |  |  |  |  |  |
| **6 months** | 0.1 ± 0.5 p=0.157 | 0.0 ± 0.4 p=<0.001 | -0.0 ± 0.5 p=1.000 |  |  |  |  |
| **12 months** | 0.1 ± 0.5 p=1.000 | -0.1 ± 0.5 p=1.000 | -0.1 ± 0.5 p=1.000 | -0.1 ± 0.5 p=1.000 |  |  |  |
| **18 months** | 0.2 ± 0.4 p=<0.001 | 0.0 ± 0.4 p=1.000 | 0.0 ± 0.4 p=1.000 | 0.1 ± 0.5 p=1.000 | 0.1 ± 0.4 p=0.186 |  |  |
| **24 months** | 0.2 ± 0.4 p=0.011 | -0.0 ± 0.4 p=1.000 | 0.0 ± 0.4  p=1.000 | 0.0 ± 0.4 p=1.000 | 0.1 ± 0.4 p=1.000 | -0.1 ± 0.3 p=1.000 |  |

**Supplementary Table 9. Pairwise Comparison of GAD-7 scores.**

Post-hoc pairwise comparisons between time points were performed using paired t-tests with Bonferroni-Holm correction for multiple comparisons. Effect sizes were calculated using Cohen's *d* for paired differences.

SD, standard deviation

| **Follow-up Interval** | **Mean Difference ± SD, p-value** | | | | | | |
| --- | --- | --- | --- | --- | --- | --- | --- |
|  | **Baseline** | **1 month** | **3 months** | **6 months** | **12 months** | **18 months** | **24 months** |
| **Baseline** |  |  |  |  |  |  |  |
| **1 month** | -2.6 ± 5.6 p=<0.001 |  |  |  |  |  |  |
| **3 months** | -2.3 ± 6.0 p=0.006 | 0.3 ± 6.4 p=1.000 |  |  |  |  |  |
| **6 months** | -2.6 ± 6.4 p=0.002 | 0.0 ± 5.6 p=1.000 | -0.4 ± 5.4 p=1.000 |  |  |  |  |
| **12 months** | -2.0 ± 7.7 p=0.196 | 0.6 ± 6.7 p=1.000 | 0.2 ± 7.9 p=1.000 | 0.6 ± 7.8 p=1.000 |  |  |  |
| **18 months** | -4.3 ± 5.7 p=<0.001 | -1.7 ± 5.6 p=0.059 | -2.0 ± 5.5  p=0.007 | -1.7 ± 4.7 p=0.012 | -2.3 ± 6.6 p=0.017 |  |  |
| **24 months** | -1.7 ± 8.2 p=0.918 | 0.9 ± 7.5 p=1.000 | 0.6 ± 8.7 p=1.000 | 1.0 ± 7.8 p=1.000 | 0.4 ± 8.6 p=1.000 | 2.6 ± 7.8 p=0.021 |  |

**Supplementary Table 10. Pairwise Comparison of SQS scores.**

Post-hoc pairwise comparisons between time points were performed using paired t-tests with Bonferroni-Holm correction for multiple comparisons. Effect sizes were calculated using Cohen's *d* for paired differences.

SD, standard deviation

| **Follow-up Interval** | **Mean Difference ± SD, p-value** | | | | | | |
| --- | --- | --- | --- | --- | --- | --- | --- |
|  | **Baseline** | **1 month** | **3 months** | **6 months** | **12 months** | **18 months** | **24 months** |
| **Baseline** |  |  |  |  |  |  |  |
| **1 month** | 1.5 ± 3.0 p=<0.001 |  |  |  |  |  |  |
| **3 months** | 0.8 ± 3.5 p=0.604 | -0.7 ± 3.0 p=0.338 |  |  |  |  |  |
| **6 months** | 1.0 ± 3.6 p=0.209 | -0.5 ± 3.8 p=1.000 | 0.2 ± 3.9 p=1.000 |  |  |  |  |
| **12 months** | 1.2 ± 4.1 p=0.116 | -0.3 ± 4.2 p=1.000 | 0.4 ± 4.5 p=1.000 | 0.2 ± 4.0 p=1.000 |  |  |  |
| **18 months** | 0.9 ± 4.2 p=0.832 | -0.6 ± 3.9 p=1.000 | 0.1 ± 4.0 p=1.000 | -0.1 ± 3.8 p=1.000 | -0.3 ± 4.4 p=1.000 |  |  |
| **24 months** | 0.5 ± 3.6 p=1.000 | -1.0 ± 3.5 p=0.144 | -0.2 ± 3.8 p=1.000 | -0.4 ± 3.8 p=1.000 | -0.6 ± 4.1 p=1.000 | -0.3 ± 3.4 p=1.000 |  |

**Supplementary Table 11. Pairwise Comparison of BPI Severity scores.**

Post-hoc pairwise comparisons between time points were performed using paired t-tests with Bonferroni-Holm correction for multiple comparisons. Effect sizes were calculated using Cohen's *d* for paired differences.

SD, standard deviation

| **Follow-up Interval** | **Mean Difference ± SD, p-value** | | | | | | |
| --- | --- | --- | --- | --- | --- | --- | --- |
|  | **Baseline** | **1 month** | **3 months** | **6 months** | **12 months** | **18 months** | **24 months** |
| **Baseline** |  |  |  |  |  |  |  |
| **1 month** | -1.0 ± 2.0 p=<0.001 |  |  |  |  |  |  |
| **3 months** | -1.5 ± 2.1  p=<0.001 | -0.5 ± 2.7 p=1.000 |  |  |  |  |  |
| **6 months** | -0.7 ± 2.8 p=0.390 | 0.3 ± 3.4  p=1.000 | 0.9 ± 3.0 p=0.120 |  |  |  |  |
| **12 months** | -1.1 ± 2.9 p=0.008 | -0.0 ± 3.1 p=1.000 | 0.5 ± 3.2  p=1.000 | -0.4 ± 3.1 p=1.000 |  |  |  |
| **18 months** | -1.2 ± 3.0 p=<0.001 | -0.2 ± 2.9 p=1.000 | 0.3 ± 3.6  p=1.000 | -0.5 ± 3.6 p=1.000 | -0.2 ± 2.6 p=1.000 |  |  |
| **24 months** | -1.5 ± 2.7 p=1.000 | -0.5 ± 3.3 p=1.000 | 0.0 ± 3.1 p=1.000 | -0.9 ± 3.2 p=0.200 | -0.5 ± 3.3 p=1.000 | -0.3 ± 4.3 p=1.000 |  |

**Supplementary Table 12. Pairwise Comparison of BPI Interference scores.**

Post-hoc pairwise comparisons between time points were performed using paired t-tests with Bonferroni-Holm correction for multiple comparisons. Effect sizes were calculated using Cohen's *d* for paired differences.

SD, standard deviation

| **Follow-up Interval** | **Mean Difference ± SD, p-value** | | | | | | |
| --- | --- | --- | --- | --- | --- | --- | --- |
|  | **Baseline** | **1 month** | **3 months** | **6 months** | **12 months** | **18 months** | **24 months** |
| **Baseline** |  |  |  |  |  |  |  |
| **1 month** | -1.4 ± 2.9 p=<0.001 |  |  |  |  |  |  |
| **3 months** | -2.0 ± 2.7 p=<0.001 | -0.6 ± 3.5 p=1.000 |  |  |  |  |  |
| **6 months** | -1.4 ± 3.6 p=0.005 | 0.1 ± 4.1 p=1.000 | 0.7 ± 3.8 p=1.000 |  |  |  |  |
| **12 months** | 1.8 ± 3.5 p=<0.001 | -0.3 ± 3.9 p=1.000 | 0.3 ± 3.9 p=1.000 | -0.4 ± 4.8 p=1.000 |  |  |  |
| **18 months** | -2.2 ± 3.8 p=<0.001 | -0.7 ± 4.1 p=1.000 | -0.1 ± 4.4  p=1.000 | -0.8 ± 4.7 p=1.000 | -0.4 ± 4.3 p=1.000 |  |  |
| **24 months** | -2.4 ± 4.4 p=<0.001 | -1.0 ± 4.6 p=0.655 | -0.4 ± 4.4 p=1.000 | -1.1 ± 5.2 p=0.799 | -0.7 ± 4.9 p=1.000 | -0.3 ± 5.0 p=1.000 |  |

**Supplementary Table 13. Pairwise Comparison of SF-MPQ-2 continuous pain subscale scores**

Post-hoc pairwise comparisons between time points were performed using paired t-tests with Bonferroni-Holm correction for multiple comparisons. Effect sizes were calculated using Cohen's *d* for paired differences.

SD, standard deviation

| **Follow-up Interval** | **Mean Difference ± SD, p-value** | | | | | | |
| --- | --- | --- | --- | --- | --- | --- | --- |
|  | **Baseline** | **1 month** | **3 months** | **6 months** | **12 months** | **18 months** | **24 months** |
| **Baseline** |  |  |  |  |  |  |  |
| **1 month** | -1.2 ± 2.6 p=<0.001 |  |  |  |  |  |  |
| **3 months** | -1.8 ± 2.8 p=<0.001 | -0.6 ± 2.8 p=0.775 |  |  |  |  |  |
| **6 months** | -1.3 ± 3.4 p=0.005 | -0.1 ± 3.7 p=1.000 | 0.5 ± 3.6 p=1.000 |  |  |  |  |
| **12 months** | -1.3 ± 3.0 p=0.001 | -0.1 ± 3.4 p=1.000 | 0.5 ± 3.5 p=1.000 | -0.0 ± 3.3 p=1.000 |  |  |  |
| **18 months** | -2.2 ± 3.1 p=<0.001 | -1.0 ± 3.4  p=0.068 | -0.4 ± 3.6  p=1.000 | -0.9 ± 3.8 p=0.324 | -0.9 ± 4.2 p=0.746 |  |  |
| **24 months** | -1.6 ± 3.3 p=<0.001 | -0.5 ± 3.7 p=1.000 | 0.1 ± 4.0 p=1.000 | -0.4 ± 4.2 p=1.000 | -0.3 ± 4.4 p=1.000 | 0.6 ± 3.6 p=1.000 |  |

**Supplementary Table 14. Pairwise Comparison of SF-MPQ-2 intermittent pain subscale scores**

Post-hoc pairwise comparisons between time points were performed using paired t-tests with Bonferroni-Holm correction for multiple comparisons. Effect sizes were calculated using Cohen's *d* for paired differences.

SD, standard deviation

| **Follow-up Interval** | **Mean Difference ± SD, p-value** | | | | | | |
| --- | --- | --- | --- | --- | --- | --- | --- |
|  | **Baseline** | **1 month** | **3 months** | **6 months** | **12 months** | **18 months** | **24 months** |
| **Baseline** |  |  |  |  |  |  |  |
| **1 month** | -1.3 ± 2.7 p=<0.001 |  |  |  |  |  |  |
| **3 months** | -1.6 ± 3.0 p=<0.001 | -0.3 ± 3.2  p=1.000 |  |  |  |  |  |
| **6 months** | -0.7 ± 3.3 p=0.781 | 0.6 ± 3.1  p=1.000 | 0.9 ± 3.4 p=0.166 |  |  |  |  |
| **12 months** | -0.3 ± 3.8 p=1.000 | 1.0 ± 4.0 p=0.236 | 1.3 ± 4.1 p=0.029 | 0.4 ± 3.9 p=1.000 |  |  |  |
| **18 months** | -1.5 ± 3.4 p=<0.001 | -0.2 ± 3.8 p=1.000 | 0.2 ± 3.9  p=1.000 | -0.8 ± 4.2 p=1.000 | -1.2 ± 3.8 p=0.033 |  |  |
| **24 months** | -1.3 ± 3.6 p=0.011 | 0.0 ± 3.9 p=1.000 | 0.3 ± 3.9 p=1.000 | -0.6 ± 4.2 p=1.000 | -1.0 ± 4.4 p=0.464 | 0.2 ± 4.5 p=1.000 |  |

**Supplementary Table 15. Pairwise Comparison of SF-MPQ-2 neuropathic pain subscale scores**

Post-hoc pairwise comparisons between time points were performed using paired t-tests with Bonferroni-Holm correction for multiple comparisons. Effect sizes were calculated using Cohen's *d* for paired differences.

SD, standard deviation

| **Follow-up Interval** | **Mean Difference ± SD, p-value** | | | | | | |
| --- | --- | --- | --- | --- | --- | --- | --- |
|  | **Baseline** | **1 month** | **3 months** | **6 months** | **12 months** | **18 months** | **24 months** |
| **Baseline** |  |  |  |  |  |  |  |
| **1 month** | -0.3 ± 2.0 p=1.000 |  |  |  |  |  |  |
| **3 months** | -0.3 ± 2.5 p=1.000 | 0.0 ± 2.1 p=1.000 |  |  |  |  |  |
| **6 months** | 0.1 ± 2.9 p=1.000 | 0.4 ± 2.5 p=1.000 | 0.4 ± 2.7 p=1.000 |  |  |  |  |
| **12 months** | -0.1 ± 2.6 p=1.000 | 0.3 ± 2.8 p=1.000 | 0.2 ± 2.8 p=1.000 | -0.1 ± 3.4 p=1.000 |  |  |  |
| **18 months** | -0.5 ± 3.0  p=1.000 | -0.2 ± 3.1 p=1.000 | -0.2 ± 3.1  p=1.000 | -0.6 ± 3.8 p=1.000 | -0.5 ± 3.3 p=1.000 |  |  |
| **24 months** | -0.5 ± 3.3 p=1.000 | -0.1 ± 3.4 p=1.000 | -0.2 ± 3.5 p=1.000 | -0.5 ± 3.8 p=1.000 | -0.4 ± 3.0 p=1.000 | 0.1 ± 3.2 p=1.000 |  |

**Supplementary Table 16. Pairwise Comparison of SF-MPQ-2 Total scores**

Post-hoc pairwise comparisons between time points were performed using paired t-tests with Bonferroni-Holm correction for multiple comparisons. Effect sizes were calculated using Cohen's *d* for paired differences.

SD, standard deviation

| **Follow-up Interval** | **Mean Difference ± SD, p-value** | | | | | | |
| --- | --- | --- | --- | --- | --- | --- | --- |
|  | **Baseline** | **1 month** | **3 months** | **6 months** | **12 months** | **18 months** | **24 months** |
| **Baseline** |  |  |  |  |  |  |  |
| **1 month** | -1.1 ± 2.0 p=<0.001 |  |  |  |  |  |  |
| **3 months** | 1.6 ± 2.3 p=<0.001 | -0.6 ± 2.2 p=0.237 |  |  |  |  |  |
| **6 months** | -0.9 ± 3.1 p=0.136 | 0.2 ± 3.2 p=1.000 | 0.8 ± 3.3 p=0.541 |  |  |  |  |
| **12 months** | -1.3 ± 2.9 p=<0.001 | -0.2 ± 2.6 p=1.000 | 0.4 ± 2.7 p=1.000 | -0.4 ± 4.1 p=1.000 |  |  |  |
| **18 months** | -1.8 ± 3.0 p=<0.001 | -0.7 ± 3.0 p=1.000 | -0.1 ± 2.6  p=1.000 | -0.9 ± 4.0 p=0.670 | -0.5 ± 2.9 p=1.000 |  |  |
| **24 months** | -1.7 ± 3.3 p=<0.001 | -0.6 ± 3.1 p=1.000 | -0.0 ± 3.1 p=1.000 | -0.8 ± 4.0 p=1.000 | -0.4 ± 3.3 p=1.000 | 0.1 ± 3.1 p=1.000 |  |

**Supplementary Table 17. Pairwise Comparison of Pain VAS scores.**

Post-hoc pairwise comparisons between time points were performed using paired t-tests with Bonferroni-Holm correction for multiple comparisons. Effect sizes were calculated using Cohen's *d* for paired differences.

SD, standard deviation

| **Follow-up Interval** | **Mean Difference ± SD, p-value** | | | | | | |
| --- | --- | --- | --- | --- | --- | --- | --- |
|  | **Baseline** | **1 month** | **3 months** | **6 months** | **12 months** | **18 months** | **24 months** |
| **Baseline** |  |  |  |  |  |  |  |
| **1 month** | -1.5 ± 3.3 p=<0.001 |  |  |  |  |  |  |
| **3 months** | -2.0 ± 3.1 p=<0.001 | -0.6 ± 3.3  p=1.000 |  |  |  |  |  |
| **6 months** | -1.0 ± 3.9 p=0.295 | 0.5 ± 3.8 p=1.000 | 1.1 ± 4.2 p=0.280 |  |  |  |  |
| **12 months** | -0.8 ± 3.5 p=0.521 | 0.7 ± 4.2  p=1.000 | 1.2 ± 4.1 p=0.063 | 0.2 ± 4.2 p=1.000 |  |  |  |
| **18 months** | -1.8 ± 3.9 p=<0.001 | -0.4 ± 4.4 p=1.000 | 0.2 ± 4.6  p=1.000 | -0.9 ± 4.4 p=0.012 | -1.0 ± 4.5 p=0.492 |  |  |
| **24 months** | -1.4 ± 4.1 p=0.024 | 0.1 ± 5.1  p=1.000 | 0.7 ± 5.0 p=1.000 | -0.4 ± 4.7 p=1.000 | -0.6 ± 4.5 p=1.000 | 0.5 ± 3.8 p=1.000 |  |

**Supplementary Table 18. Proportion of individuals who reported an MCID / improvement in each PrOM at 1, 3, 6, 12, 18 and 24 months.**

N, number of participants; %, percentage

| **PrOM** | **MCID Improvement** | **1 month**  N (%) | **3 months**  N (%) | **6 months**  N (%) | **12 months**  N (%) | **18 months**  N (%) | **24 months**  N (%) |
| --- | --- | --- | --- | --- | --- | --- | --- |
| **EQ-5D-5L Index** | **0** | 27 (26.7) | 24 (23.8) | 37 (36.6) | 37 (36.6) | 30 (29.7) | 35 (34.7) |
|  | **1** | 74 (73.3) | 77 (76.2) | 64 (63.4) | 64 (63.4) | 71 (70.3) | 66 (65.4) |
| **GAD-7** | **0** | 67 (66.3) | 60 (59.4) | 57 (56.4) | 57 (56.4) | 45 (44.6) | 57 (56.4) |
|  | **1** | 34 (33.7) | 41 (40.6) | 44 (43.6) | 44 (43.6) | 56 (55.5) | 44 (43.6) |
| **SQS** | **0** | 65 (64.4) | 71 (70.3) | 68 (67.3) | 63 (62.4) | 62 (61.4) | 69 (68.3) |
|  | **1** | 36 (35.6) | 30 (29.7) | 33 (32.7) | 38 (37.6) | 39 (38.6) | 32 (31.7) |
| **BPI Severity** | **0** | 58 (57.4) | 50 (49.5) | 57 (56.4) | 49 (48.5) | 50 (49.5) | 46 (45.5) |
|  | **1** | 43 (42.6) | 51 (50.5) | 44 (43.6) | 52 (51.5) | 51 (50.5) | 55 (54.5) |
| **BPI Interference** | **0** | 48 (47.5) | 39 (38.6) | 50 (49.5) | 46 (45.5) | 39 (38.6) | 42 (41.6) |
|  | **1** | 53 (52.5) | 62 (61.4) | 51 (50.5) | 55 (54.5) | 62 (61.4) | 59 (58.4) |
| **SF-MPQ-2 Total** | **0** | 52 (51.5) | 43 (42.6) | 51 (50.5) | 44 (43.6) | 37 (36.6) | 40 (39.6) |
|  | **1** | 49 (48.5) | 58 (57.4) | 50 (49.5) | 57 (56.4) | 64 (63.4) | 61 (60.4) |
| **Pain VAS** | **0** | 37 (36.6) | 36 (35.6) | 52 (51.5) | 53 (52.5) | 40 (39.6) | 47 (46.5) |
|  | **1** | 64 (63.4) | 65 (64.4) | 49 (48.5) | 48 (47.5) | 61 (60.4) | 54 (53.5) |

**Supplementary Table 19: Results of univariate logistic regression analyses evaluating factors a minimal clinically important difference in the Brief Pain Inventory-Short Form (BPI) Severity at 24 months.** Variables assessed include gender, age, body mass index (BMI), cannabis status, cannabis-based medicinal products (CBMPs) at 24 months, cannabidiol (CBD) dose at 24 months, (−)-trans-Δ^9^-tetrahydrocannabinol (THC) dose at 24 months, baseline Sleep Quality Scale (SQS), and baseline Generalised Anxiety Disorder-7 (GAD-7) scores.

| Variable | N | Odds Ratio  [95% Confidence Interval] | p-value |
| --- | --- | --- | --- |
| **Age Category:**  Under 30  31-40  Over 40 | 29  45  27 | -  0.7 [0.3-1.9]  0.8 [0.3-2.3] | Ref  0.506  0.629 |
| **BMI Category:**  Under 20  20-24.99  25-29.99  30 and over | 13  34  23  27 | 0.9 [0.2-3.4]  -  0.7 [0.2-2.1]  0.7 [0.2-1.9] | 0.840  Ref  0.534  0.468 |
| **Cannabis Status:**  Never used  Current user  Ex-user | 29  42  30 | -  0.6 [0.2-1.5]  0.4 [0.1-1.1] | Ref  0.266  0.081 |
| **CBMP Category:**  Oil  Dried flower/ flos  Dried flow/ flos, Oil  Other | 22  19  52  8 | -  2.3 [0.6-9.3]  1.2 [0.4-3.4]  0.8 [0.2-4.4] | Ref  0.209  0.686  0.825 |
| **CBD category:**  Minimum to Q1  Q1 to Median  Median to Q3  Q3 to Maximum | 27  24  26  24 | -  0.9 [0.3-2.8]  2.1 [0.7-6.6]  2.3 [0.7-7.5] | Ref  0.895  0.199  0.169 |
| **THC category:**  Minimum to Q1  Q1 to Median  Median to Q3  Q3 to Maximum | 26  25  26  24 | -  1.5 [0.5-4.6]  1.6 [0.5-4.9]  2.4 [0.8-8.2] | Ref  0.474  0.403  0.137 |
| **SQS Score:**  0-3  4-6  7-10 | 36  39  26 | 1.7 [0.6-4.8]  0.9 [0.3-2.3]  - | 0.342  0.760  Ref |
| **GAD-7 Score:**  Less than 5  5-9  10-14  15 and over | 31  35  17  18 | -  1.1 [0.4-2.9]  1.2 [0.4-4.0]  2.9 [0.8-12.0] | Ref  0.851  0.790  0.115 |

**Supplementary Table 20: Results of multivariate logistic regression analyses evaluating factors a minimal clinically important difference in the Brief Pain Inventory-Short Form (BPI) Severity at 24 months.** Variables assessed include gender, age, body mass index (BMI), cannabis status, cannabis-based medicinal products (CBMPs) at 24 months, cannabidiol (CBD) dose at 24 months, (−)-trans-Δ9-tetrahydrocannabinol (THC) dose at 24 months, baseline Sleep Quality Scale (SQS), and baseline Generalised Anxiety Disorder-7 (GAD-7) scores.

| Variable | N | Odds Ratio  [95% Confidence Interval] | p-value |
| --- | --- | --- | --- |
| **Age Category:**  Under 30  31-40  Over 40 | 27  44  26 | -  1.3 [0.4-4.6]  2.0 [0.5-8.6] | Ref  0.686  0.357 |
| **BMI Category:**  Under 20  20-24.99  25-29.99  30 and over | 13  34  23  27 | 1.6 [0.3-9.7]  -  1.7 [0.4-7.5]  0.6 [0.2-2.3] | 0.607  Ref  0.458  0.480 |
| **Cannabis Status:**  Never used  Current user  Ex-user | 26  41  30 | -  1.0 [0.3-4.0]  0.4 [0.1-1.5] | Ref  0.991  0.165 |
| **CBMP Category:**  Oil  Dried flower/ flos  Dried flow/ flos, Oil  Other | 20  17  52  8 | -  0.5 [0.0-15.5]  0.2 [0.0-3.7]  0.1 [0.0-2.5] | Ref  0.672  0.304  0.151 |
| **CBD category:**  Minimum to Q1  Q1 to Median  Median to Q3  Q3 to Maximum | 27  23  25  22 | -  0.4 [0.1-1.9]  2.2 [0.5-10.3]  0.9 [0.2-3.9] | Ref  0.243  0.304  0.830 |
| **THC category:**  Minimum to Q1  Q1 to Median  Median to Q3  Q3 to Maximum | 24  24  26  23 | -  1.5 [0.1-47.0]  9.5 [0.6-298.3]  3.9 [0.3-121.8] | Ref  0.775  0.126  0.356 |
| **SQS Score:**  0-3  4-6  7-10 | 36  35  26 | 1.2 [0.3-4.6]  0.3 [0.1-1.0]  - | 0.778  0.065  Ref |
| **GAD-7 Score:**  Less than 5  5-9  10-14  15 and over | 30  33  17  17 | -  2.9 [0.8-11.9]  2.6 [0.6-12.7]  3.7 [0.7-22.3] | Ref  0.116  0.209  0.134 |

**Supplementary Table 21: Results of univariate logistic regression analyses evaluating factors a minimal clinically important difference in the Brief Pain Inventory-Short Form (BPI) Interference at 24 months.** Variables assessed include gender, age, body mass index (BMI), cannabis status, cannabis-based medicinal products (CBMPs) at 24 months, cannabidiol (CBD) dose at 24 months, (−)-trans-Δ9-tetrahydrocannabinol (THC) dose at 24 months, baseline Sleep Quality Scale (SQS), and baseline Generalised Anxiety Disorder-7 (GAD-7) scores.

| Variable | N | Odds Ratio [95% Confidence Interval] | p-value |
| --- | --- | --- | --- |
| **Age Category:**  Under 30  31-40  Over 40 | 29  45  27 | -  0.9 [0.3-2.3]  0.9 [0.3-2.6] | Ref  0.795  0.817 |
| **BMI Category:**  Under 20  20-24.99  25-29.99  30 and over | 13  34  23  27 | 1.6 [0.4-6.8]  -  0.6 [0.2-1.9]  1.2 [0.4-3.4] | 0.513  Ref  0.415  0.742 |
| **Cannabis Status:**  Never used  Current user  Ex-user | 29  42  30 | -  1.9 [0.7-5.0]  0.8 [0.3-2.3] | Ref  0.208  0.698 |
| **CBMP Category:**  Oil  Dried flower/ flos  Dried flow/ flos, Oil  Other | 22  19  52  8 | -  1.1 [0.3-3.9]  1.7 [0.6-4.8]  0.6 [0.1-3.1] | Ref  0.867  0.283  0.546 |
| **CBD category:**  Minimum to Q1  Q1 to Median  Median to Q3  Q3 to Maximum | 27  24  26  24 | -  0.7 [0.2-2.1]  0.8 [0.3-2.4]  0.6 [0.2-1.8] | Ref  0.525  0.695  0.352 |
| **THC category:**  Minimum to Q1  Q1 to Median  Median to Q3  Q3 to Maximum | 26  25  26  24 | -  2.5 [0.8-8.0]  2.2 [0.7-6.9]  1.0 [0.3-3.0] | Ref  0.119  0.166  0.982 |
| **SQS Score:**  0-3  4-6  7-10 | 36  39  26 | 2.0 [0.7-5.7]  1.1 [0.4-2.9]  - | 0.189  0.919  Ref |
| **GAD-7 Score:**  Less than 5  5-9  10-14  15 and over | 31  35  17  18 | -  1.3 [0.5-3.3]  1.1 [0.3-3.5]  1.9 [0.6-6.6] | Ref  0.653  0.930  0.307 |

**Supplementary Table 22: Results of multivariate logistic regression analyses evaluating factors a minimal clinically important difference in the Brief Pain Inventory-Short Form (BPI) Interference at 24 months.** Variables assessed include gender, age, body mass index (BMI), cannabis status, cannabis-based medicinal products (CBMPs) at 24 months, cannabidiol (CBD) dose at 24 months, (−)-trans-Δ9-tetrahydrocannabinol (THC) dose at 24 months, baseline Sleep Quality Scale (SQS), and baseline Generalized Anxiety Disorder-7 (GAD-7) scores.

| Variable | N | Odds Ratio [95% Confidence Interval] | p-value |
| --- | --- | --- | --- |
| **Age Category:**  Under 30  31-40  Over 40 | 27  44  26 | -  0.2 [0.0-0.7]  0.1 [0.0-0.6] | Ref  0.018  0.013 |
| **BMI Category:**  Under 20  20-24.99  25-29.99  30 and over | 13  34  23  27 | 3.6 [0.5-40.0]  -  0.4 [0.1-1.7]  1.5 [0.4-5.8] | 0.212  Ref  0.220  0.585 |
| **Cannabis Status:**  Never used  Current user  Ex-user | 26  41  30 | -  0.9 [0.2-3.5]  1.5 [0.3-6.4] | Ref  0.872  0.607 |
| **CBMP Category:**  Oil  Dried flower/ flos  Dried flow/ flos, Oil  Other | 20  17  52  8 | -  0.1 [0.0-5.0]  0.8 [0.0-18.3]  0.1 [0.0-6.4] | Ref  0.295  0.863  0.310 |
| **CBD category:**  Minimum to Q1  Q1 to Median  Median to Q3  Q3 to Maximum | 27  23  25  22 | -  0.7 [0.1-4.9]  0.2 [0.0-0.7]  0.7 [0.1-3.6] | Ref  0.751  0.022  0.622 |
| **THC category:**  Minimum to Q1  Q1 to Median  Median to Q3  Q3 to Maximum | 24  24  26  23 | -  12.2 [0.6-498.9]  39.5 [1.9-1892.3]  10.4 [0.6-415.0] | Ref  0.128  0.032  0.153 |
| **SQS Score:**  0-3  4-6  7-10 | 36  35  26 | 4.7 [1.2-22.0]  1.5 [0.4-6.5]  - | 0.037  0.581  Ref |
| **GAD-7 Score:**  Less than 5  5-9  10-14  15 and over | 30  33  17  17 | -  0.3 [0.1-1.1]  1.6 [0.3-9.3]  1.2 [0.2-7.9] | Ref  0.075  0.606  0.867 |

**Supplementary Table 23: Results of univariate logistic regression analyses evaluating factors a minimal clinically important difference in the Short-Form McGill Pain Questionnaire – Version 2 (SF-MPQ 2).** Variables assessed include gender, age, body mass index (BMI), cannabis status, cannabis-based medicinal products (CBMPs) at 24 months, cannabidiol (CBD) dose at 24 months, (−)-trans-Δ9-tetrahydrocannabinol (THC) dose at 24 months, baseline Sleep Quality Scale (SQS), and baseline Generalised Anxiety Disorder-7 (GAD-7) scores.

| Variable | N | Odds Ratio [95% Confidence Interval] | p-value |
| --- | --- | --- | --- |
| **Age Category:**  Under 30  31-40  Over 40 | 29  45  27 | -  0.5 [0.2-1.4]  0.4 [0.1-1.2] | Ref  0.177  0.108 |
| **BMI Category:**  Under 20  20-24.99  25-29.99  30 and over | 13  34  23  27 | 3.0 [0.8-15.0]  -  1.7 [0.6-5.1]  5.1 [1.6-20.3] | 0.144  Ref  0.359  0.011 |
| **Cannabis Status:**  Never used  Current user  Ex-user | 29  42  30 | -  1.0 [0.3-2.6]  1.5 [0.5-4.5] | Ref  0.915  0.515 |
| **CBMP Category:**  Oil  Dried flower/ flos  Dried flow/ flos, Oil  Other | 22  19  52  8 | -  2.1 [0.6-9.5]  1.0 [0.3-2.8]  1.7 [0.3-13.6] | Ref  0.287  0.989  0.562 |
| **CBD category:**  Minimum to Q1  Q1 to Median  Median to Q3  Q3 to Maximum | 27  24  26  24 | -  0.9 [0.2-3.0]  0.5 [0.1-1.5]  0.7 [0.2-2.4] | Ref  0.796  0.211  0.563 |
| **THC category:**  Minimum to Q1  Q1 to Median  Median to Q3  Q3 to Maximum | 26  25  26  24 | -  1.4 [0.4-4.6]  1.0 [0.3-3.2]  1.1 [0.3-3.5] | Ref  0.611  1.000  0.924 |
| **SQS Score:**  0-3  4-6  7-10 | 36  39  26 | 0.5 [0.2-1.5]  0.9 [0.3-2.8]  - | 0.234  0.910  Ref |
| **GAD-7 Score:**  Less than 5  5-9  10-14  15 and over | 31  35  17  18 | -  0.5 [0.2-1.3]  1.6 [0.4-8.4]  0.6 [0.2-1.9] | Ref  0.150  0.522  0.341 |

**Supplementary Table 24: Results of multivariate logistic regression analyses evaluating factors a minimal clinically important difference in the Short-Form McGill Pain Questionnaire – Version 2 (SF-MPQ 2).** Variables assessed include gender, age, body mass index (BMI), cannabis status, cannabis-based medicinal products (CBMPs) at 24 months, cannabidiol (CBD) dose at 24 months, (−)-trans-Δ9-tetrahydrocannabinol (THC) dose at 24 months, baseline Sleep Quality Scale (SQS), and baseline Generalised Anxiety Disorder-7 (GAD-7) scores.

| Variable | N | Odds Ratio [95% Confidence Interval] | p-value |
| --- | --- | --- | --- |
| **Age Category:**  Under 30  31-40  Over 40 | 27  44  26 | -  0.3 [0.1-1.3]  0.5 [0.1-2.2] | Ref  0.131  0.327 |
| **BMI Category:**  Under 20  20-24.99  25-29.99  30 and over | 13  34  23  27 | 4.8 [0.9-32.1]  -  2.7 [0.7-11.6]  7.9 [1.8-44.7] | 0.081  0.158  Ref  0.010 |
| **Cannabis Status:**  Never used  Current user  Ex-user | 26  41  30 | -  0.5 [0.2-2.1]  1.3 [0.3-6.0] | Ref  0.379  0.720 |
| **CBMP Category:**  Oil  Dried flower/ flos  Dried flow/ flos, Oil  Other | 20  17  52  8 | -  10.3 [0.3-558.4]  3.2 [0.1-124.6]  9.0 [0.2-654.0] | Ref  0.208  0.482  0.275 |
| **CBD category:**  Minimum to Q1  Q1 to Median  Median to Q3  Q3 to Maximum | 27  23  25  22 | -  1.4 [0.3-8.2]  0.3 [0.1-1.4]  0.7 [0.1-3.4] | Ref  0.695  0.132  0.631 |
| **THC category:**  Minimum to Q1  Q1 to Median  Median to Q3  Q3 to Maximum | 24  24  26  23 | -  0.4 [0.0-7.2]  0.5 [0.0-10.1]  0.7 [0.0-12.7] | Ref  0.533  0.680  0.775 |
| **SQS Score:**  0-3  4-6  7-10 | 36  35  26 | 0.8 [0.2-3.2]  1.1 [0.2-4.6]  - | 0.731  0.931  Ref |
| **GAD-7 Score:**  Less than 5  5-9  10-14  15 and over | 31  33  16  17 | -  0.2 [0.0-0.7]  1.9 [0.3-17.3]  0.3 [0.1-1.8] | Ref  0.021  0.520  0.207 |

**Supplementary Table 25: Results of univariate logistic regression analyses evaluating factors a minimal clinically important difference in the Visual Analogue Scale (VAS).** Variables assessed include gender, age, body mass index (BMI), cannabis status, cannabis-based medicinal products (CBMPs) at 24 months, cannabidiol (CBD) dose at 24 months, (−)-trans-Δ9-tetrahydrocannabinol (THC) dose at 24 months, baseline Sleep Quality Scale (SQS), and baseline Generalised Anxiety Disorder-7 (GAD-7) scores.

| Variable | N | Odds Ratio [95% Confidence Interval] | p-value |
| --- | --- | --- | --- |
| **Age Category:**  Under 30  31-40  Over 40 | 29  45  27 | -  0.5 [0.2-1.2]  0.8 [0.3-2.3] | Ref  0.629  1.000 |
| **BMI Category:**  Under 20  20-24.99  25-29.99  30 and over | 13  34  23  27 | 1.6 [0.4-6.2]  -  1.1 [0.4-3.2]  1.7 [0.6-4.9] | 0.480  -  0.872  0.313 |
| **Cannabis Status:**  Never used  Current user  Ex-user | 29  42  30 | -  2.5 [0.9-6.7]  1.2 [0.4-3.5] | Ref  0.070  0.691 |
| **CBMP Category:**  Oil  Dried flower/ flos  Dried flow/ flos, Oil  Other | 22  19  52  8 | -  0.5 [0.1-1.8]  0.5 [0.2-1.5]  0.3 [0.1-1.5] | Ref  0.311  0.256  0.140 |
| **CBD category:**  Minimum to Q1  Q1 to Median  Median to Q3  Q3 to Maximum | 27  24  26  24 | -  1.3 [0.4-4.2]  0.9 [0.3-2.8]  0.8 [0.3-2.4] | Ref  0.615  0.901  0.692 |
| **THC category:**  Minimum to Q1  Q1 to Median  Median to Q3  Q3 to Maximum | 26  25  26  24 | -  0.8 [0.3-2.4]  0.5 [0.2-1.4]  1.0 [0.3-3.3] | Ref  0.688  0.168  0.944 |
| **SQS Score:**  0-3  4-6  7-10 | 36  39  26 | 1.4 [0.5-3.9]  1.3 [0.5-3.5]  - | 0.516  0.612  Ref |
| **GAD-7 Score:**  Less than 5  5-9  10-14  15 and over | 31  35  17  18 | -  1.0 [0.4-2.8]  4.5 [1.3-19.0]  6.9 [1.8-34.5] | Ref  0.940  0.026  0.008 |

**Supplementary Table 26: Results of multivariate logistic regression analyses evaluating factors a minimal clinically important difference in the Pain Visual Analogue Scale (VAS).** Variables assessed include gender, age, body mass index (BMI), cannabis status, cannabis-based medicinal products (CBMPs) at 24 months, cannabidiol (CBD) dose at 24 months, (−)-trans-Δ9-tetrahydrocannabinol (THC) dose at 24 months, baseline Sleep Quality Scale (SQS), and baseline Generalised Anxiety Disorder-7 (GAD-7) scores.

| Variable | N | Odds Ratio [95% Confidence Interval] | p-value |
| --- | --- | --- | --- |
| **Age Category:**  Under 30  31-40  Over 40 | 27  44  26 | -  0.3 [0.1-1.0]  0.3 [0.1-1.4] | Ref  0.055  0.134 |
| **BMI Category:**  Under 20  20-24.99  25-29.99  30 and over | 13  34  23  27 | 1.6 [0.3-9.7]  -  1.4 [0.4-5.9]  4.0 [1.1-18.0] | 0.622  Ref  0.601  0.052 |
| **Cannabis Status:**  Never used  Current user  Ex-user | 26  41  30 | -  3.3 [0.9-14.0]  1.7 [0.4-7.2] | Ref  0.089  0.456 |
| **CBMP Category:**  Oil  Dried flower/ flos  Dried flow/ flos, Oil  Other | 20  17  52  8 | -  0.0 [0.0-0.4]  0.0 [0.0-0.7]  0.0 [0.0-0.5] | Ref  0.026  0.047  0.029 |
| **CBD category:**  Minimum to Q1  Q1 to Median  Median to Q3  Q3 to Maximum | 27  23  25  22 | -  0.4 [0.1-2.1]  2.2 [0.5-11.1]  2.0 [0.4-9.9] | Ref  0.294  0.301  0.407 |
| **THC category:**  Minimum to Q1  Q1 to Median  Median to Q3  Q3 to Maximum | 24  24  26  23 | -  7.4 [0.4-357.1]  5.1 [0.3-222.8]  9.6 [0.5-468.2] | Ref  0.234  0.319  0.175 |
| **SQS Score:**  0-3  4-6  7-10 | 36  35  26 | 1.2 [0.3-4.9]  1.0 [0.3-3.5]  - | 0.762  0.965  Ref |
| **GAD-7 Score:**  Less than 5  5-9  10-14  15 and over | 31  33  16  17 | -  1.3 [0.4-4.7]  5.3 [1.2-28.8]  10.5 [1.8-88.6] | Ref  0.668  0.040  0.017 |

**Supplementary Figure 1: Pairwise analysis of pain specific patient-reported outcome measures (PrOMs) (n=101). A) Brief Pain Inventory – Short Form (BPI) Severity; B) BPI Interference; C) Short Form – McGill Pain Questionnaire – 2 (SF-MPQ-2) Total Score; D) Pain Visual Analogue Scale (VAS)**
